# The human microbiota is a beneficial reservoir for SARS-CoV-2 mutations

**DOI:** 10.1128/mbio.03187-23

**Published:** 2024-03-26

**Authors:** Birong Cao, Xiaoxi Wang, Wanchao Yin, Zhaobing Gao, Bingqing Xia

**Affiliations:** 1 State Key Laboratory of Drug Research, Shanghai Institute of Materia Medica, Chinese Academy of Sciences, Shanghai, China; 2 University of Chinese Academy of Sciences, Beijing, China; 3 Guangdong Guangya High School, Guangzhou, China; 4 Zhongshan Institute for Drug Discovery, Shanghai Institute of Materia Medica, Chinese Academy of Sciences, Zhongshan, China; University of Calgary, Calgary, Canada

**Keywords:** human microbiota, SARS-CoV-2 advantageous mutations, evolution

## Abstract

Severe acute respiratory syndrome coronavirus 2 (SARS-CoV-2) mutations are rapidly emerging. In particular, beneficial mutations in the spike (S) protein, which can either make a person more infectious or enable immunological escape, are providing a significant obstacle to the prevention and treatment of pandemics. However, how the virus acquires a high number of beneficial mutations in a short time remains a mystery. We demonstrate here that variations of concern may be mutated due in part to the influence of the human microbiome. We searched the National Center for Biotechnology Information database for homologous fragments (HFs) after finding a mutation and the six neighboring amino acids in a viral mutation fragment. Among the approximate 8,000 HFs obtained, 61 mutations in S and other outer membrane proteins were found in bacteria, accounting for 62% of all mutation sources, which is 12-fold higher than the natural variable proportion. A significant proportion of these bacterial species—roughly 70%—come from the human microbiota, are mainly found in the lung or gut, and share a composition pattern with COVID-19 patients. Importantly, SARS-CoV-2 RNA-dependent RNA polymerase replicates corresponding bacterial mRNAs harboring mutations, producing chimeric RNAs. SARS-CoV-2 may collectively pick up mutations from the human microbiota that change the original virus’s binding sites or antigenic determinants. Our study clarifies the evolving mutational mechanisms of SARS-CoV-2.

**IMPORTANCE:**

Severe acute respiratory syndrome coronavirus 2 (SARS-CoV-2) mutations are rapidly emerging, in particular advantageous mutations in the spike (S) protein, which either increase transmissibility or lead to immune escape and are posing a major challenge to pandemic prevention and treatment. However, how the virus acquires a high number of advantageous mutations in a short time remains a mystery. Here, we provide evidence that the human microbiota is a reservoir of advantageous mutations and aids mutational evolution and host adaptation of SARS-CoV-2. Our findings demonstrate a conceptual breakthrough on the mutational evolution mechanisms of SARS-CoV-2 for human adaptation. SARS-CoV-2 may grab advantageous mutations from the widely existing microorganisms in the host, which is undoubtedly an “efficient” manner. Our study might open a new perspective to understand the evolution of virus mutation, which has enormous implications for comprehending the trajectory of the COVID-19 pandemic.

## INTRODUCTION

As of October 2022, 32,000 amino acid (aa) mutations had been found in the proteins of the severe acute respiratory syndrome coronavirus 2 (SARS-CoV-2) (https://ngdc.cncb.ac.cn) (1, 2). Mutations in the spike (S) protein were of particular significance because the S protein mediates viral entry into a host cell and is a target for vaccine development (3–5). Certain S mutations can change how well a virus enters human cells and increase transmissibility by 10–100 times. These mutations include N501Y, D614G, and L452R (6–10). Mutations E484K, F490L, G446S, K417N, L452R, N501Y, and S477N, located in the receptor-binding domain (RBD) of the S protein, significantly reduce the effects of neutralizing antibodies and vaccine efficacy (4, 11, 12). These mutations, which either increase transmissibility or lead to immune escape, are thought to be beneficial mutations to SARS-CoV-2. Based on altered clinical disease presentation or elevated transmissibility, mostly due to mutations in the S protein, nine variants have been designated as variants of concern (VOCs) by the World Health Organization: the Alpha variant (originally identified in the UK, B.1.1.7), the Beta variant (originally identified in South Africa, B.1.351), the Gamma variant (originally identified in Brazil, P.1), the Delta variant (originally identified in India, B.1.617.2), and the Omicron variant (originally identified in India, BA.1–BA.5) (13, 14). The COVID-19 pandemic has expanded into new regions due to the swift generation of mutations, which presents a significant obstacle to the efficacy of treatment drugs and vaccinations (15, 16).

According to some research, SARS-CoV-2 evolution and natural selection will inevitably lead to the acquisition of advantageous mutations (17, 18). Nonetheless, it is noteworthy that the VOCs seem to have gained the extraordinary ability to spread around the world in a short time. Being an enveloped positive-sense, single-stranded RNA virus, SARS-CoV-2 requires an RNA-dependent RNA polymerase (RdRp) for replication, which is inherently prone to error (3). However, this type of replication-associated mutation is not easy to obtain via evolutionarily beneficial mutation, and although they benefit a virus by promoting host adaptation capacity, they can be fatal to the virus itself (6, 17). Recombination among coronaviruses or across viral species may lead to a greater number of beneficial mutations than acquired through replication, leading to significant changes in the phenotype of SARS-CoV-2 (19, 20). Only a small number of recombination events, primarily involving this variation and other coronavirus subspecies in the same genus, have been discovered thus far, making it difficult to understand how SARS-CoV-2 acquired a high number of beneficial mutations in a short time (18, 21).

A number of peculiar “events” contribute to the enigma surrounding SARS-CoV-2’s mutational development. On the one hand, some VOCs share identical dominant mutations, such as N501Y and D164G in the Alpha, Beta, Gamma, and Omicron variants and K417N and L452R in the Delta and Omicron variants, supporting the evolutionary relationships of these VOCs (6, 22). Nevertheless, certain mutations developed simultaneously in distinct geographic places. For example, N501Y appeared simultaneously in South Africa and England (23, 24). Finally, compared to other VOCs, the Omicron variant is unique. Omicron carries 25 unique mutations in the S protein, which suggests that some of these mutations were not derived from variants circulating before and, therefore, might have a different origin. The Omicron was a perfect virus to employ for examining (25–27). One hypothesis suggests that the Omicron variant may have “cryptically spread” for a long time and circulated in a population with insufficient viral surveillance and sequencing, suggesting a similar evolutionary mechanism to the other VOCs (28, 29). Another hypothesis suggests that the Omicron variant may have evolved in immunocompromised COVID-19 patients, supporting an intrahost evolution during prolonged infection (30–32). The sequence of the Omicron S protein significantly coincides with that of SARS-CoV-2, which has mutations known to enhance adaptation in mice, suggesting, finally, that the Omicron variation may have accrued mutations in a nonhuman host (33, 34).

Nevertheless, SARS-CoV-2 rapidly gained several advantageous mutations, which aided in its evolution toward enhanced adaptability and allowed it to evolve nearly perfectly in that direction, as evidenced by a number of indicators. However, the current hypotheses and theories are insufficient to explain how SARS-CoV-2 acquired a high number of mutations, particularly those with evolutionary advantages, which have enabled the virus to spread in the face of increasing population immunity while maintaining or increasing replication fitness. Therefore, unrecognized factors have facilitated SARS-CoV-2 evolution into a better adaptive species. These factors would be expected to exhibit the following traits if they were responsible for the rapid mutation: widely distributed in hosts, contributing to easy viral access to hosts, and being immunologically tolerant to hosts. The potential factors that facilitate beneficial mutation are explored. Here, we designed a fragment-based blast approach and identified the homologous fragments (HFs) containing advantageous mutations in the outer membrane proteins of SARS-CoV-2. It was found that human microbiota might be the major host contributing to the viral mutation acquisition.

## RESULTS

### A viral mutation fragment-based blast was developed to identify homologous fragments harboring beneficial mutations

**Fig 1:**
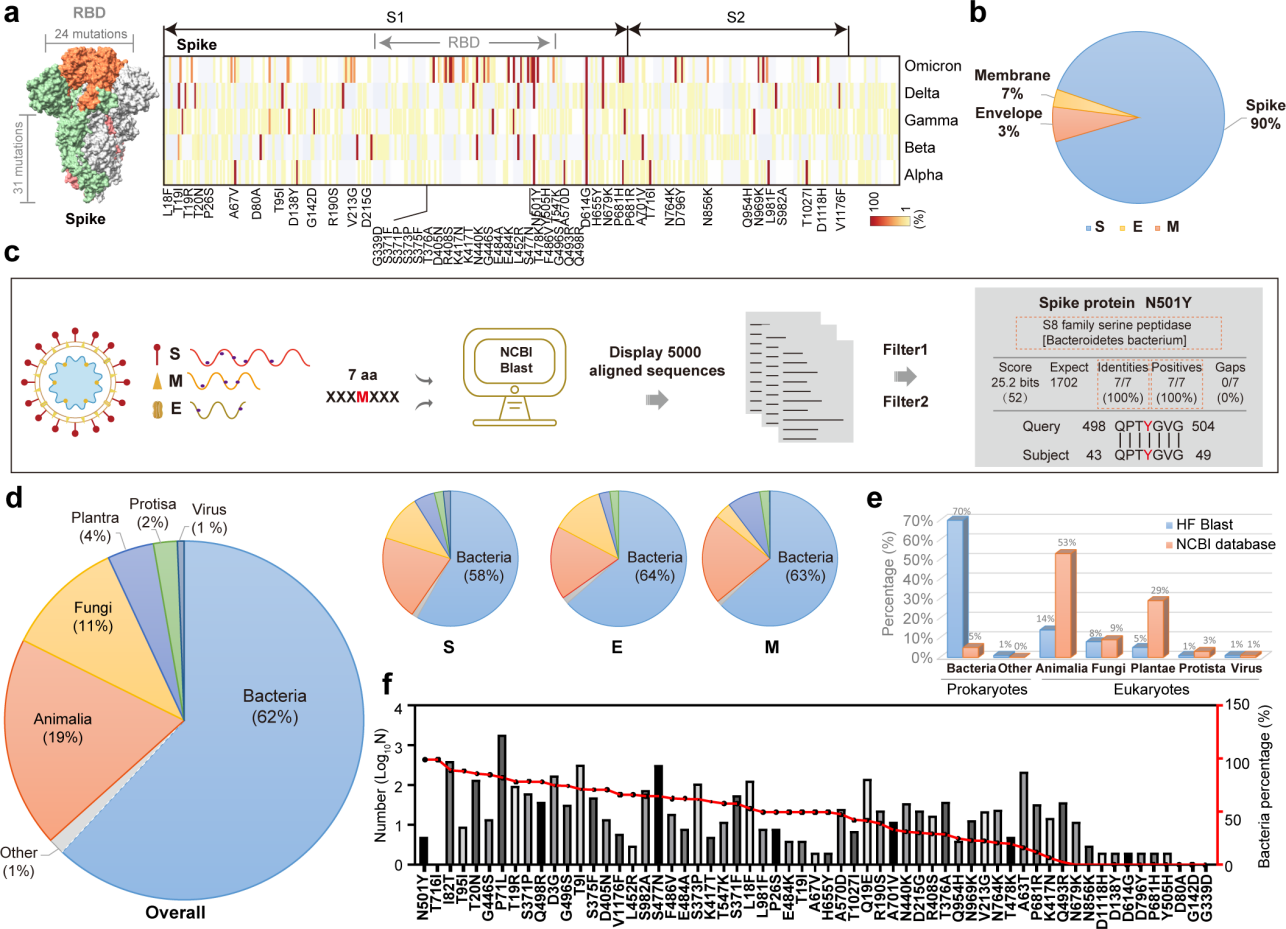
Homologous fragment alignment of viral mutation fragments harboring high-frequency mutations in outer membrane proteins. (**a)** Structure of the SARS-CoV-2 S protein. The RBD domain containing 24 mutations is marked in orange (left). The probability of S protein mutation in all VOCs (right).** (b)** The ratio of high-frequency mutations in the spike (**S**), envelope (E), and membrane (**M**) proteins. (**c),** A flow chart showing homologous sequence alignment of viral mutation fragments in the NCBI database. (**d)** Pie chart representations of HF kingdoms derived from overall (left bigger), spike (**S**), envelope (**e**), and membrane (**M**) (right smaller three) mutations. (**e)** The species proportions contributing HFs and in NCBI databases. (**f)** Correlation analysis of the number of HFs for each mutation and the corresponding bacterial contribution.

The first thing that was examined was the frequency of S protein mutations in all VOCs. We found that among the identified 3,116 S protein mutations, all 55 beneficial mutations had a mutation frequency higher than 90% (1, 2). The S protein comprises two domains: the S1 domain, which contains the RBD, and the S2 domain, which is critical for membrane fusion (5). The RBD mediates attachment to human cells and is the primary target of neutralizing antibodies, and it carries 90% of S mutations, including the intensively studied N501Y, E484K, L452Q, and F486V (Fig. 1a) (12, 35). In addition, some mutations in addition to those of the S protein may markedly affect the direction of the pandemic; these include T9I, a mutation in the membrane envelope (E) protein, leading to virulence reduction in the Omicron variant (36). This finding inspired us to include the high-frequency mutations in E and membrane (M) proteins, two other structural membrane proteins constituting the outer protein envelope and S protein together. Ultimately, a total of 61 mutations were identified: 55 S protein mutations, 2 E protein mutations, and 4 M protein mutations (Fig. 1b; Fig. S1a). Notably, the Omicron variants, including BA.4 and BA.5 subvariants, carry 38 mutations, which is a much higher number than that of any other VOCs (Fig. S1; Table S1).

We concentrated on the relevant viral mutation fragment (VMF), which consists of a mutant amino acid and its neighboring amino acids as a unit, as opposed to a single amino acid mutation alone. A previous study showed that PRRA, a VMF containing four aa, functioned as a furin cleavage site (37–39). The length of a VMF was tentatively set to be five aa, and a VMF-based blast, aiming to identify homologous fragments of these VMFs, was carried out with the NCBI Protein database, the largest protein database. We found that when the length of VMFs was five aa, numerous HFs were obtained for many VMFs. When the length of VMFs was extended to seven aa, approximately 97% of queries led to identical subjects. When the length was further extended to 9 or 11 aa, less than 7% of the queries led to matches. Therefore, VMFs containing seven aa (XXXMXXX) were selected for the original analysis database creation, with “M” indicating one mutation and “X” indicating the adjacent aa (Table S2). Among the subjects obtained, only those fulfilling all of the following criteria were collected: (i) 100% VMF identity, that is, the seven aa was fully recognized in the database; (ii) 100% homologous sequences; and (iii) sequence origins from SARS proteins were ignored. At this stage, we obtained approximately 8,000 subjects in total. To prevent duplicative statistics and overcome the limitation of the lower error, we defined two filter conditions: (i) for multiple accessions for one protein, only one accession was retained, and (ii) for multiple isoforms for the same protein, only one isoform was retained. After filtering, 5,600 subjects were collected and were used to establish the final database for analysis, and it included the name, ID number, species, and submission number for each HF (Fig. 1c; Tables S3-1, S3-2, S3-3, and S4).

### Bacteria contributed more than 60% of the homologous fragments

The species proportion in the final database was analyzed. We were astonished to find that bacteria contributed more than 60% of the HFs. At the same time, Animalia, Fungi, Plantae, and Protista accounted for 19%, 11%, 4%, and 2%, respectively (Fig. 1d). Notably, of the 742,923 subjects in the NCBI protein database, more than 93.5% were found in eukaryotes. Fewer than 5% were found in bacteria (Fig. 1e; Fig. S2). The markedly inverse proportion inspired us to pay close attention to the bacteria. The species proportions were further analyzed based on individual mutation location, and no significant difference was found among S, E, and M protein mutations (Fig. 1d). Then, the database was analyzed from the perspective of individual mutations. We found that the number of HFs ranged from 0 to 2,075, averaging 92 for each of the 61 evaluated mutations. No HFs were found for three mutations (D80A, G142D, and G339D), accounting for only 7% of the total evaluated mutations. Interestingly, although bacterial HFs were identified for 93% of the mutations, for each mutation, the bacterial proportion was not correlated with the number of HFs. For certain mutations, both the identified HF and bacterial contributions were very high; these mutations included S447N (209), T20N (119), T76I (264), and P71L (1,523). In contrast, fewer than five HFs were found for some mutations, such as N501Y (5), L452R (3), and T716I (1), but all of the HFs were from bacteria. Notably, the bacterial proportion for N679K was 0%, and for the other six mutations, although the corresponding HF number was high, it was 13, arguing against the idea that the bacterial contribution that was revealed was a coincidence (Fig. 1f).

### The human microbiota is a reservoir of homologous fragments

**Fig 2:**
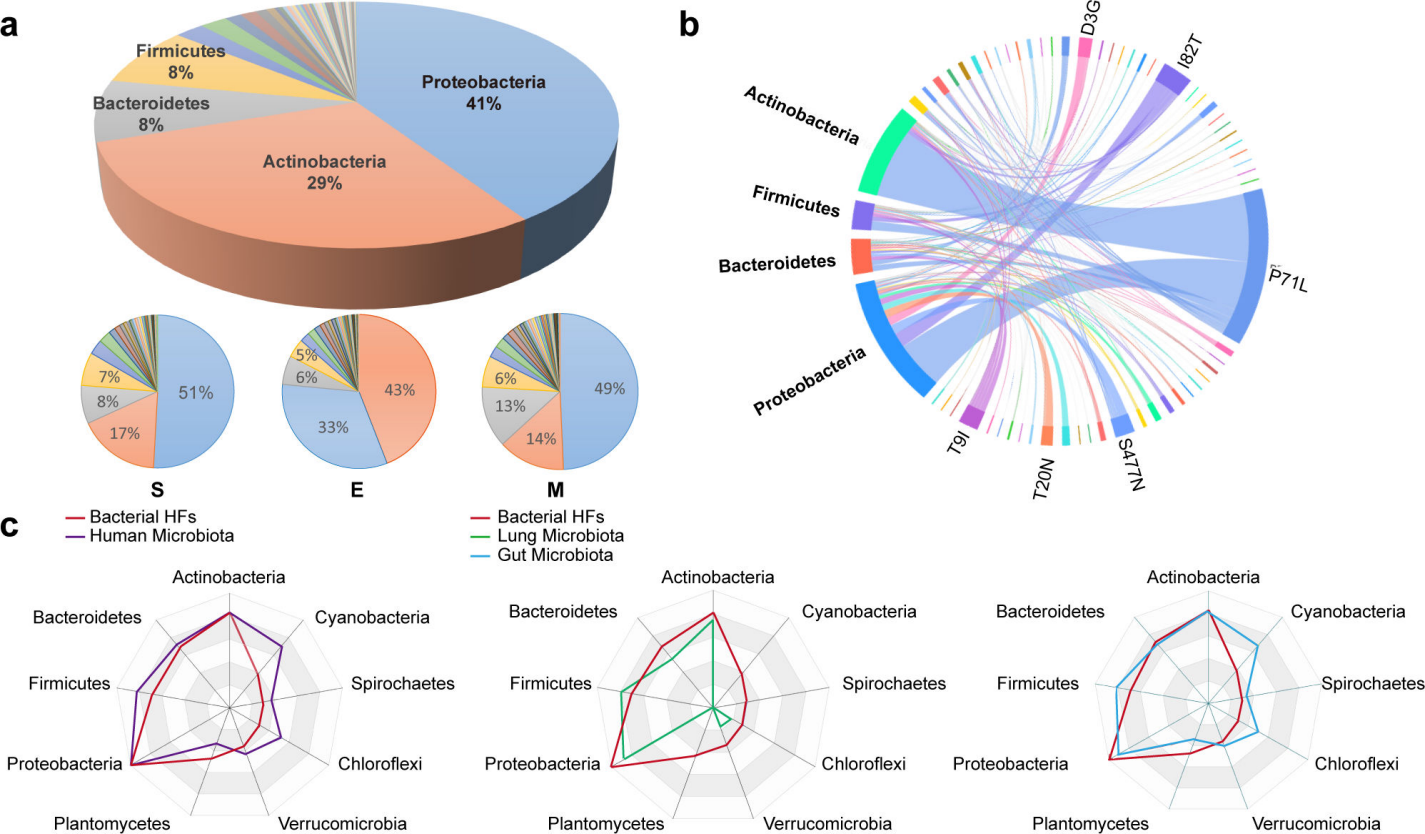
Homologous fragment composition analysis of the obtained bacteria. (**a)** Pie chart representations of the obtained bacteria, all items (up) and items of spike, envelope, and membrane proteins (bottom) at the phylum level. (b) Chord diagram displaying the network of mutated HFs and their bacterial phyla. (**c)** Radar maps showing the correlation of the identified bacteria sources of HFs (red), human microbiota (purple) (top), lung microbiota (green) (below left), and gut microbiota (cyan) (below right) at the phylum level. In detail, we collected information on the types and respective counts of genera within the top nine bacterial phyla from our study samples. In parallel, we obtained the types and quantity of human microbiota, gut microbiota, and lung microbiota from the database. The collected data were processed to calculate the proportions of different bacterial phyla and genera. Through Venn comparison, the common bacteria were identified. Then, we calculated the proportion of these common bacteria in each phylum.

Next, through categorical and quantitative analysis, we attempted to comprehend the makeup of the microorganisms. Based on phylogeny and taxonomy theories, the 3,271 obtained bacterial species belonged to 73 different phyla. Despite the diversity of these phyla, most of the bacterial species obtained were classified into four major bacterial phyla, namely, Proteobacteria, 41%; Actinobacteria, 29%; Bacteroidetes, 8%, and Firmicutes, 8% (Fig. 2a). Notably, the four major phyla containing HFs were consistent with the four major phyla of human microbiota, although the proportions were different (40, 41). The composition of the bacterial species with S, M, and E protein mutations was then analyzed individually, and a composition pattern similar to that of the mutations in total was obtained. The correlations between the mutations and bacterial phyla with proportions greater than 1% were plotted. We found that all 61 mutations matched multiple bacterial phyla (Fig. 2b). For example, HFs carrying I82T were found in descending order in Proteobacteria, Bacteroidetes, Firmicutes, and Actinobacteria, while HFs containing T9I were found in descending order in Bacteroidetes, Actinobacteria, and Firmicutes. In addition, some bacterial phyla carried multiple HFs, including Proteobacteria, which contributed more than 51 different types of HFs (Fig. 2b). To deepen our understanding of the composition of the contributing bacteria, core species were further categorized at the family and genus levels. The four major bacterial phyla contained 331 families, including 132 in Proteobacteria, 89 in Actinobacteria, 30 in Firmicutes, and 28 in Bacteroidetes (Fig. S3a). At the genus level, Streptomyces and genera in the Pseudomonadaceae, Enterobacteriaceae, Micromonospora, Xanthomonadaceae, Rhizobiaceae, Burkholderiaceae, Flavobacteriaceae, and Comamonadaceae families showed average HF abundances of ≥1%, and therefore, they were considered to be the top 10 HF-contributing genera (Fig. S3b).

The human body contains trillions of microorganisms that infect the skin, oral cavity, intestines, and even the lungs, making it the biggest host of SARS-CoV-2 (42–45). The four main phyla between the human microbiota and the bacteria were identical, enabling deep correlation analysis. As shown in the radar chart, almost all bacteria belonged to the human microbiota at the phylum level. In COVID-19 patients, the lung is a major organ affected by viral infection or viral-bacterial coinfection. Approximately 56% of the bacteria we obtained are found in the lungs of healthy individuals; these include Streptomycetaceae and Staphylococcaceae (Fig. 2c) (42, 46). Although bacteria in the lungs are abundant, microbial cell populations reach their highest density in the intestines, where they form the gut microbiota and are believed to be important to human life (43, 44). Interestingly, 83% of the obtained bacterial species were also found in the human gut microbiota database (Fig. 2c), implying the importance of the gut microbiota.

### The species composition of the homologous fragments is similar to that of the human gut microbiota in COVID-19 patients

**Fig 3:**
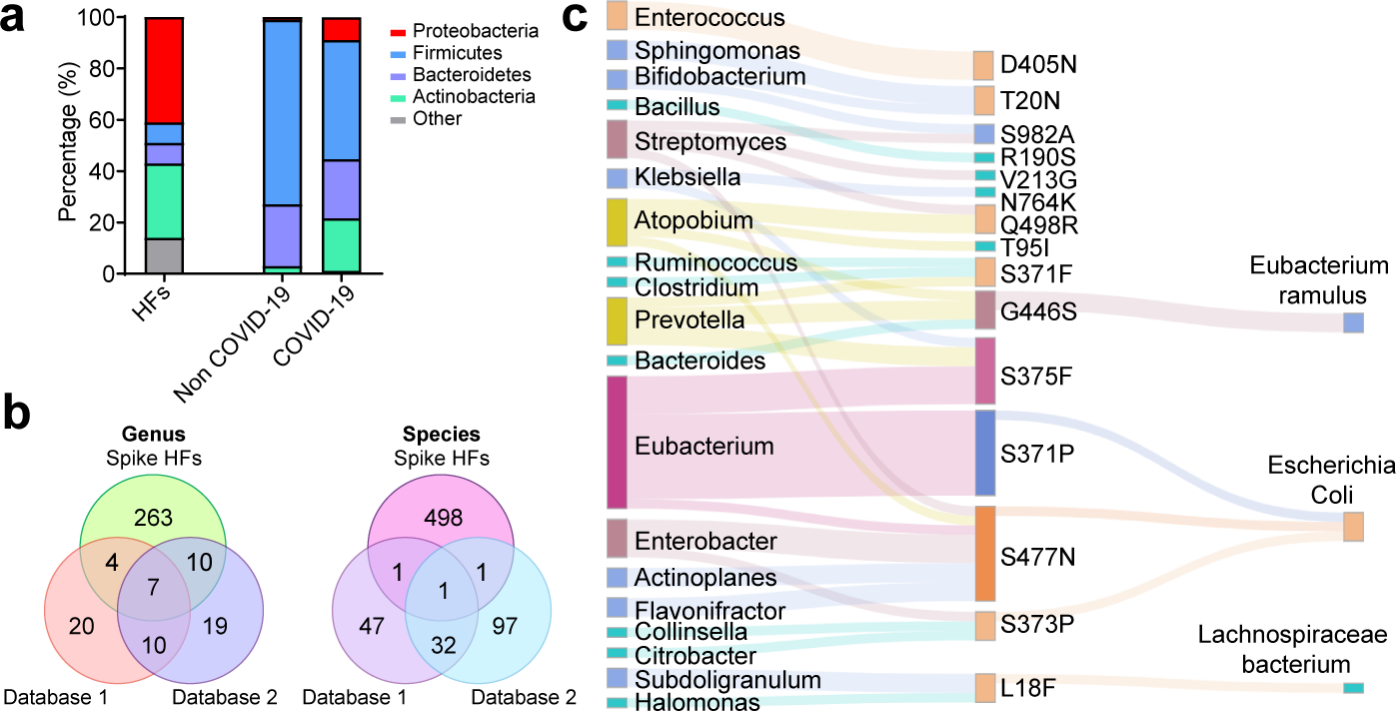
Correlation of the identified bacteria carrying homologous fragments and those in the human gut microbiota in COVID-19 patients. (**a)** The abundance of microbial phyla detected in HFs and stools from in-hospital patients with COVID-19 and non-COVID-19 individuals. The average relative abundances of COVID-19 and non-COVID-19 individuals were obtained from two databases (gutMEGA). The baseline proportions of the healthy human microbiome composition are Firmicutes, approximately 72%, Bacteroidetes, which constitute around 24%, Actinobacteria, approximately 2.5%, and Proteobacteria, which comprise around 0.5% of the total bacterial population. (**b)** Intersection analysis showing the obtained bacteria containing HFs with the gut microbes of COVID-19 patient databases at the genus (left) and species (right) levels. Two databases were used (gutMEGA). (**c)** A Sankey diagram showing the relationship of the mutations in the spike protein with changes in bacteria containing HFs in COVID-19 patients at the genus (left side) and species (right side) levels.

Furthermore, 10% of COVID-19 patients also had diarrhea and other gastrointestinal problems (47, 48). It has been reported that in approximately 50% of COVID-19 patients, the virus was found in feces, leading to the hypothesis that there is not only replication and, therefore, activity in the intestine but also that the virus resides for prolonged periods in the intestines (49–51). Increasing evidence suggests that gut bacteria are altered in COVID-19 patients, and these alterations are associated with infection severity, treatment effectiveness, and prognosis (52, 53). The gut microbial ecological network is significantly weakened and becomes diffuse in patients with COVID-19, and together, the number of beneficial bacteria is decreased, and harmful bacteria production multiplies (52, 54–56). Two gut microbe databases of COVID-19 patients (gutMEGA) were analyzed. Compared with the composition of non-COVID-19 gut microbes, two abnormal changes were detected in the COVID-19 entries. The population of beneficial bacteria, particularly Firmicutes, significantly declined to 47% from 72%, while that of harmful bacteria, such as Proteobacteria, increased to 9% from 1%. Notably, a similar change was observed for the obtained bacteria carrying HFs, including an increase in harmful Proteobacteria to 41% and a reduction in beneficial Firmicutes to 8% (Fig. 3a) (52, 53, 56). The influences of gut bacteria changes identified in the two databases were perhaps best exemplified by an intersection analysis with the bacteria containing S protein mutations (Fig. 3b). In the two COVID-19 patient databases, at the family level, 17 and 11 coexpressed bacteria were identified; at the species level, three coexpressed bacteria were identified. These correlations at the genus and species levels were further analyzed (Fig. 3c). At the genus level, we found that among the 55 S mutations, 15 were detected in 19 differential bacterial genera in COVID-19 patients. At the species level, three types of bacteria in COVID-19 patients were found to carry mutational HFs (Fig. 3c). Notably, one genus of bacteria, Escherichia, carried three different mutations (S371P, S373P, and S477N). These strong associations supported the hypothesis that SARS-CoV-2 might obtain HFs from the human microbiota, especially in light of the small size of the present gut bacteria database of COVID-19 patient data.

After the emergence of BA.5, various new Omicron subvariants appeared, including BA.5.2 and BF.7, two major pandemic variants, and BQ.1.1 and XBB, the most resistant SARS-CoV-2 variants discovered to date. These new recombinant subvariants carried five new beneficial mutations, as determined on the basis of the original Omicron, with mutation frequencies higher than 90%, including R346T, K444T, N460K, and F486S in the S protein and T11A in the E protein (Fig. S4a). We applied our VMF-based blast model to search for potential HFs carrying these five mutations. As expected, bacteria contributed more than 80% of the HFs with these five mutations, while Animalia, fungi, Plantae, and Protista accounted for 4%, 4%, 5%, and 1%, respectively (Fig. S4b; Table S5). Moreover, the identified bacterial species were also classified into four major bacterial phyla, namely, Firmicutes, 49%; Proteobacteria, 26%; Actinobacteria, 11%; and Bacteroidetes, 4% (Fig. S4c), which were the same as those carrying mutations previously identified.

### SARS-CoV-2 RdRp replicates bacterial mRNAs to form chimeric viral-bacterial RNAs harboring mutations

**Fig 4:**
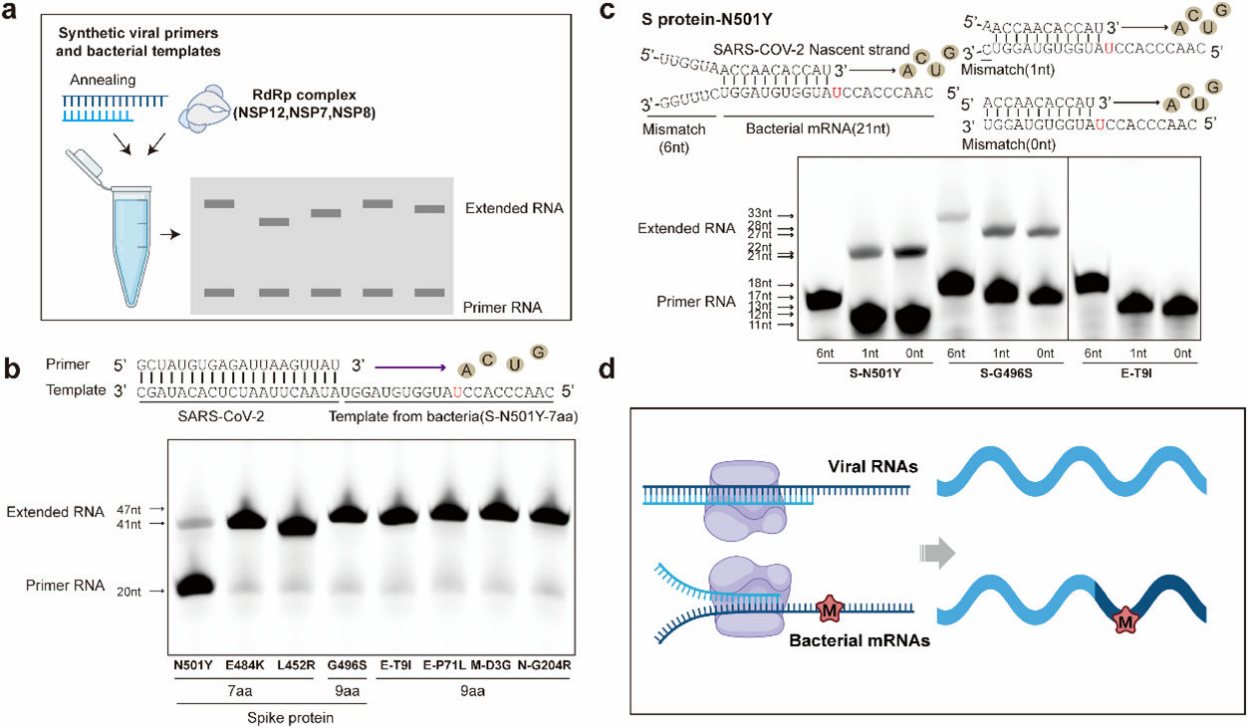
SARS-CoV-2 RdRp introduces bacterial mRNA nucleotide mutations into viral RNAs through homologous recombination. (**a)** Schematic diagram showing the RdRp extension experiment. (**b)** RNA duplex with a 5′-21 or 27 nt from bacterial mRNA overhang as the template for primer extension and RdRp-RNA complex assembly (exemplified by N501Y). The primer strand is labeled with a fluorescence molecule at the 5′ end. The gels are spliced for space conservation and to enhance the layout of the figure. The original data are shown in Fig. S6. (c) The primer strand from SARS-CoV-2 contains 11 or 12 nucleotides that complementarily pair with bacterial template mRNAs but have zero, one, or six mismatches at the 5′ end and in a duplex formation with the bacterial template mRNAs harboring N501Y, G496S, or T9I mutations for RdRp extension (exemplified by N501Y).** (d)** Schematic diagram showing RdRp introducing a nucleotide mutation into a bacterial mRNA to produce a bacterial-viral chimeric RNA via homologous recombination.

In contrast to SARS-CoV-2, bacterial HFs are encoded by mRNAs. Nucleotide sequences of bacterial mRNAs encoding the same amino acid sequences as viral RNAs were aligned, revealing a percent identity (PNI) from 63% to 90% in the seven examined fragments (Fig. S5). A PNI less than 100% can be possibly due to the degeneration of the genetic code and particularly the presence of synonymous mutations in SARS-CoV-2 (GenBank accession: NC_045512.2; Wuhan-Hu-1) (57, 58). SARS-CoV-2 replication is mediated by a multisubunit replication-and-transcription complex of viral nonstructural proteins (nsp), of which nsp12 is the core component of the RNA-dependent RNA polymerase (3). When these bacterial mRNAs introduce mutations into a viral genome, they must serve as templates for the viral RdRp complex, which was examined experimentally (Fig. 4a). The bacterial mRNAs (up to 27 nt long) carrying mutations such as N501Y, E484K, and L452R, which were fused with a previously validated viral duplex RNA, were successfully replicated by an RdRp complex containing nsp12, nsp7, and nsp8 (Fig. 4b; Table S6). This experiment demonstrated that exogenous bacterial mRNAs are effective templates for SARS-CoV-2 RdRp.

During SARS-CoV-2 replication, base pairings with 6–12 consecutive nucleotides can serve as “junction” sites for template switching, i.e., RdRp switches from copying one genome to copying another, resulting in a subgenome (59–62). Notably, under certain circumstances, such as when lesions are included in a template, replication-impeding secondary structures are formed, and the tightly bound nucleotide pool is imbalanced, template switching may also be evident among RNAs of different species (61, 63, 64). Whether the complementary base pairs between bacterial mRNA and viral RNA can act as “junction” sites was therefore examined. As shown in Fig. 4c, the nascent primer strand from SARS-CoV-2 contained 11/12/17 nucleotides; three bacterial template mRNAs harboring the N501Y mutation carried 11 nucleotides complementary to the viral primer, but zero, one, or six mismatches in the 5′ end. Among the three nucleotide groups, only the last group failed to extend. Then, two more types of bacterial template mRNAs harboring G496S (12 complementary nt) or T9I (12 complementary nt) were individually examined under identical conditions. The extension was observed for all G496S mRNAs regardless of the number of mismatches in the 5′ end, but the extension failed for all the T9I mRNAs (Fig. 4b through d; Table S6). The different replication efficiency was perhaps due to the insufficient efficacy of RdRp in the *in vitro* system. Although these results demonstrated that nucleotide mutations in bacterial mRNAs can be introduced into viral RNAs during replication via the RdRp, clear evidence showing that mutations can be integrated into the SARS-CoV-2 genome was not found. Because one cannot rule out the possibility that artificial coinfection of live viruses and bacteria in laboratories may promote the emergence of new mutations with unpredictable capabilities, experiments with viral RNA and live bacteria are currently prohibited at our organization.

**Fig 5:**
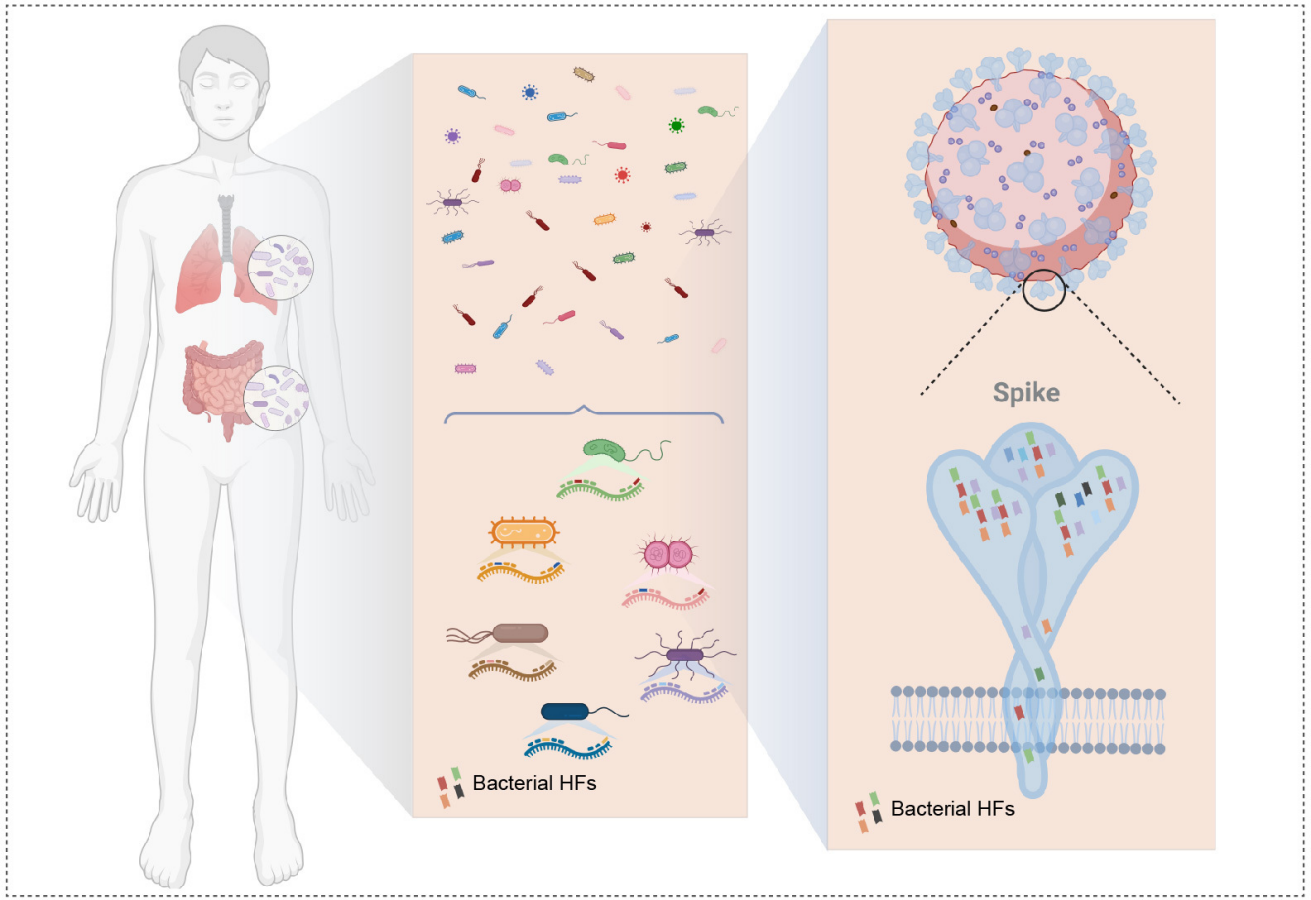
SARS-CoV-2 VOCs are chimeras containing homologous fragments from the human microbiota. Schematic graph showing SARS-CoV-2 acquiring HFs from human microbiota as exemplified by the S protein.The diagrams were created with Biorender.com.

Globally, there are now 700 million infections as of December 2022. The ultrahigh number of infections and wide regions of infection may have provided a wealth of bacterial carriers for the Omicron variant that was not available for other variants. Although data errors cannot be ruled out due to different infection density data and variable persistence of gene surveillance for different variants, all the evidence provided above supports the idea that SARS-CoV-2 may acquire HFs from the human microbiota, as is summarized in a schematic graph (Fig. 5). SARS-CoV-2 VOCs have changed from the original strain to chimeras that incorporate elements of the human microbiota.

## DISCUSSION

There is evidence to support the theory that SARS-CoV-2 has a high potential for acquiring advantageous mutations (6, 18). Here, a VMF-based blast analysis was performed to explore sources of high-frequency beneficial mutations identified in VOCs. The human microbiome accounted for about 70% of the species with HFs that were detected. In particular, the changes in the pattern of gut bacteria in COVID-19 patients are quite similar to those of the bacteria we identified harboring HFs. In addition, nucleotide mutations in bacterial mRNAs can be introduced into viral RNAs during replication via RdRp, suggesting that the human microbiome may facilitate SARS-CoV-2 mutation accumulation as a possible template reservoir.

There are at least three possibilities that could account for the viral access to bacterial mRNA in the unlikely event that SARS-CoV-2 acquires mutations from human bacteria. In Scenario 1, bacteria and viruses are both exposed to phagocytes, including macrophages and neutrophils. Bacterial coinfection is a common complication of SARS-CoV-2 infections. In particular, severely affected patients suffer a significantly higher rate of coinfection with bacteria (26%) (65). The coinfecting bacteria include mainly *Acinetobacter baumannii*, *Klebsiella pneumoniae*, and *Pseudomonas aeruginosa*, which were also identified in our study. These scenarios provide infinite possibilities for virus–bacteria interactions during the mutational evolution of SARS-CoV-2.

In light of this, during coinfection, macrophages are likely to serve as both bacterial and viral genome hosts (66–68). In Scenario 2, bacterial mRNA is exposed to nonphagocytic cells, such as infected cells or impaired cells. During SARS-CoV-2 infection, the immune barrier is impaired, which may cause invasive pathogens such as Streptococcus to break through the immune barrier and invade nonphagocytic cells (69, 70). The invaded host cells can target intracellular bacteria through autophagic machinery to lyse the bacteria and block bacterial proliferation. Nevertheless, phagocytosis by phagocytes may result from autophagy’s inability to stop the growth and multiplication of bacteria. At this point, the bacterial genome and the viral genome may coexist in host cells or phagocytes. In addition, the genetic information from the lysed bacteria may be engulfed by infected cells in some cases (71–73). In Scenario 3, some bacteria might be potential hosts for the virus. The mechanism underlying the invasion of bacteria by phages (a group of viruses that infect bacteria) has been elucidated and has become a reliable gene-editing method (74). Furthermore, some bacterial strains can raise the frequency of viral coinfection in mammalian cells, allowing for genetic recombination between two distinct viruses, eliminating harmful mutations, and regaining viral fitness (75). Recently, studies have shown that some ciliates, a type of single-cell organism, consumed virus particles, which fostered their population growth, revealing an unexpected role for viruses in ecosystems (76). As the two most abundant microbial entities, the coexistence of bacteria and viruses remains puzzling. The interactions between the virus and bacterium throughout the mutational evolution of SARS-CoV-2 are endless in these settings.

Numerous abrupt changes in the viral sequence during evolution have been found in SARS-CoV-2 reference genome analysis, indicating possible recombination events (6, 18, 21). The present study supports the idea that the complementary base pairs between bacterial and viral RNAs promote the recombination and production of chimeric RNAs harboring mutations. For viruses, homologous recombination is the most common type of recombination, including homologous recombination among viruses of the same genus and homologous recombination between viruses of different genera but with high relatedness, cross-species recombination (77–82). RNA‒RNA interactions are the determinants for the template switching efficacy among SARS-CoV-2 RNAs, with 6–12 consecutive complementary nucleotides serving as “junction” sites. The longest bacterial HF that we found was nine aa, which is noteworthy since it shows that bacterial and viral RNAs can exchange up to 27 consecutive complementary nucleotides. In a less-than-ideal *in vitro* experimental system used in the present study, we showed that 11 consecutive complementary nucleotides were sufficient to generate chimeric RNAs resulting from template switching. During homologous recombination, nucleotide mutations were simultaneously introduced into the viral chimeric RNA. Notably, not all mutations were matched to an HF (Fig. 1f). Consequently, the findings are consistent with the replication-associated mutation and evolutionary pressure selection theories, even though our investigation highlighted the significance of RdRp-mediated homologous recombination between bacterial and viral RNAs (57, 83–85). Our study speculates that bacteria may serve as potential hosts, possessing advantages such as richness, diversity, evolutionary adaptability, and immunological tolerance, which facilitate the rapid acquisition of mutational genetic material by viruses. The relationship between our study and other mutation theories requires further investigation, and we do not exclude the possibility of coexistence under different conditions.

Our work highlights the pivotal role of bacteria in facilitating viruses to acquire advantageous mutations. Bacteria offer several benefits, including their abundance, diversity, evolutionary adaptability, and tolerance to host immunity. The interaction between viral infections, especially SARS-CoV-2, and the human microbiome has garnered significant attention in recent scientific research, revealing intricate dynamics in host-pathogen interactions. Viral infection disrupts the human microbiome equilibrium in the gut, lungs, and oral cavity, influencing disease progression and viral evolution (86, 87). For instance, a shift toward microbiota with reduced short-chain fatty acid production compromises gut integrity and immune regulation (86, 88). Moreover, virus-induced changes in microbiota composition can modulate host immune responses, potentially exacerbating symptoms and complications by weakening immune defenses or creating favorable environments for viral replication (86, 88, 89). Additionally, microbiota-derived natural products exert environmental selection pressures, favoring specific viral mutations and influencing viral adaptability, such as bacteriocins affecting bacterial and viral community dynamics (90). Understanding this complex relationship not only advances infectious disease research but also underscores the importance of leveraging these interactions for innovative disease prevention and management strategies.

COVID-19 is rapidly spreading worldwide. In addition to tracking the evolution of SARS-CoV-2 from its first introduction into human populations to the present, scientists are also investigating the mechanisms underlying the acquisition of SARS-CoV-2 variations. They are tracking recombination and transmission events in the SARS-CoV-2 population in real time. Our research broadens the perspective to include virus and microbiota studies but leaves many questions unanswered, such as how and where SARS-CoV-2 access bacteria *in vivo* and how chimeric RNAs are harboring mutations integrated into the SARS-CoV-2 genome. Whether bacteria can be potential hosts in the process of virus transmission and the relationship between viruses, bacteria, and hosts deserves further exploration. Based on the examination of acquired mutations in the virus, which was not enough to account for the bidirectional causative relationship between viruses and bacteria, our conclusions were reached. In addition, due to multiple factors and experimental restrictions, we were unable to provide more experimental evidence, but we hope that more researchers in this field can perform in-depth investigations. For example, bacterial-viral co-infection models will elucidate genetic recombination mechanisms between viruses and the microbiome. Assessing long-term viral effects on the host microbiome may link these impacts to disease development and treatment response. Our research has significant ramifications for understanding the COVID-19 pandemic’s future course and creating preventive and therapeutic measures.

## MATERIALS AND METHODS

### Identification of HFs

Homologous fragments’ identification was carried out in the NCBI Protein database. VMFs (seven aa) were submitted to the NCBI protein blast research website. The alignment parameters were set as Database Non-redundant protein sequences (nr), Max target sequences 5,000, Expect threshold 0.05, and Word size 7. Among the obtained subjects, only those that fulfill all the following criteria were collected: (i) 100% VMF identity, that is, the query seven aa could be fully recognized by the database; (ii) 100% homologous sequences; and (iii) sequence origins from SARS proteins were ignored. In order to avoid duplicate statistics and overcome the limitation of the lower error, we further defined two filter conditions: (i) multiple accessions for one protein, only one accession was retained; (ii) multiple isoforms for the same protein, only one isoform was retained. After filtering, the name, ID number, species, and submission number were collected.

### Sequence data processing, inferring gut microbiota composition, and statistical analysis

The composition of the obtained HFs of bacteria was determined through categorization and quantitative analysis through the NCBI database and the UniProt database. Following this, microbiota composition profiles were inferred from quality-filtered forward reads using MetaPhlAn218 V.2.7.7 with the V.20 database. Associations of HFs’ microbial species with human microbiota parameters were identified using gutMEGA, GMrepo, China National GeneBank Microbiome, Human Oral Microbiome Database V3, and NIH Human Microbiome Project.

### RNA extension assays

All RNA oligonucleotides (templates and primer) were chemically synthesized by Genscript, in which the primer had a FAM-labeled (5(6)-carboxyfluorescein) at the 5′ end. Template and primer oligonucleotides were annealed at a 1:1 molar ratio by a 5-min heating at 85°C followed by gradually cooling to room temperature in the annealing buffer (10 mM Tris-HCl, pH 8.0, 2.5 mM EDTA, and 25 mM NaCl). The primer sequences are listed in Table S6.

RNA extension reactions contained annealed RNA (2.7 µM), nsp12 (2.7 µM), nsp7 (6.75 µM), and nsp8 (6.75 µM) in reaction buffer (20 mM Tris, pH 8.0, 10 mM KCl, 6 mM MgCl_2_, 0.01% Triton-X100, and 1 mM DTT) with 10 mM NTP (2.5 mM ATP, 2.5 mM UTP, 2.5 mM GTP, and 2.5 mM CTP) and 1.14 U/µL RNA inhibitor. The total volume of the reaction solution was 20 µL. After incubating at 25°C for 1.5 h, 40 µL quench buffer (94% formamide and 30 mM EDTA, prepared with DEPC-treated water) was added to stop the extension reaction. The sample was prepared with 8 µL reaction solution and 2 µL 5× DNA loading buffer and then loaded onto a 15% Urea-PAGE denatured gel, run at 90 V for 1.5 h, and visualized by Typhoon 95000 FLA Imager.

## SUPPLEMENTAL MATERIAL

Table S1 mbio.03187-23-s0001.xlsx Frequency of all mutations in 5 VOC variants.

Table S2 mbio.03187-23-s0002.xlsx The HFs sequences of spike, envelope, and membranes proteins.

Table S3-1 mbio.03187-23-s0003.xlsx Original data for HFs on spike protein.

Table S3-2 mbio.03187-23-s0004.xlsx Original data for HFs on envelope protein,.

Table S3-3 mbio.03187-23-s0005.xlsx Original data for HFs on membrane protein.

Table S4 mbio.03187-23-s0006.xlsx Original data for 9aa HFs on spike, envelope, and membranes proteins.

Supplemental material mbio.03187-23-s0007.docx Fig. S1-S6 and captions to supplemental tables.

Table S5 mbio.03187-23-s0008.xlsx Original data for HFs on Omicron subvariants, including BA.5.2, BF.7, BQ.1.1, and XBB.

Table S6 mbio.03187-23-s0009.xlsx The primer list for RdRp extension assays.

ASM does not own the copyrights to Supplemental Material that may be linked to, or accessed through, an article. The authors have granted ASM a non-exclusive, world-wide license to publish the Supplemental Material files. Please contact the corresponding author directly for reuse.

## References

[1] Song S, Ma L, Zou D, Tian D, Li C, Zhu J, Chen M, Wang A, Ma Y, Li M, et al.. 2020. The global landscape of SARS-CoV-2 genomes, variants, and haplotypes in 2019nCoVR. Genom Proteom Bioinform 18:749–759. doi:10.1016/j.gpb.2020.09.001 PMC7836967 33704069

[2] CNCB-NGDC Members and Partners. 2022. Database resources of the national genomics data center, China national center for bioinformation in 2022. Nucleic Acids Res 50:D27–D38. doi:10.1093/nar/gkab951 34718731 PMC8728233

[3] V’kovski P, Kratzel A, Steiner S, Stalder H, Thiel V. 2021. Coronavirus biology and replication: implications for SARS-CoV-2. Nat Rev Microbiol 19:155–170. doi:10.1038/s41579-020-00468-6 33116300 PMC7592455

[4] Yang H, Rao Z. 2021. Structural biology of SARS-CoV-2 and implications for therapeutic development. Nat Rev Microbiol 19:685–700. doi:10.1038/s41579-021-00630-8 34535791 PMC8447893

[5] Jackson CB, Farzan M, Chen B, Choe H. 2022. Mechanisms of SARS-CoV-2 entry into cells. Nat Rev Mol Cell Biol 23:3–20. doi:10.1038/s41580-021-00418-x 34611326 PMC8491763

[6] Tao K, Tzou PL, Nouhin J, Gupta RK, de Oliveira T, Kosakovsky Pond SL, Fera D, Shafer RW. 2021. The biological and clinical significance of emerging SARS-CoV-2 variants. Nat Rev Genet 22:757–773. doi:10.1038/s41576-021-00408-x 34535792 PMC8447121

[7] Plante JA, Liu Y, Liu J, Xia H, Johnson BA, Lokugamage KG, Zhang X, Muruato AE, Zou J, Fontes-Garfias CR, Mirchandani D, Scharton D, Bilello JP, Ku Z, An Z, Kalveram B, Freiberg AN, Menachery VD, Xie X, Plante KS, Weaver SC, Shi P-Y. 2021. Spike mutation D614G alters SARS-CoV-2 fitness. Nature 592:116–121. doi:10.1038/s41586-020-2895-3 33106671 PMC8158177

[8] Korber B, Fischer WM, Gnanakaran S, Yoon H, Theiler J, Abfalterer W, Hengartner N, Giorgi EE, Bhattacharya T, Foley B, Hastie KM, Parker MD, Partridge DG, Evans CM, Freeman TM, de Silva TI, McDanal C, Perez LG, Tang H, Moon-Walker A, Whelan SP, LaBranche CC, Saphire EO, Montefiori DC, Sheffield COVID-19 Genomics Group. 2020. Tracking changes in SARS-CoV-2 spike: evidence that D614G increases infectivity of the COVID-19 virus. Cell 182:812–827. doi:10.1016/j.cell.2020.06.043 32697968 PMC7332439

[9] Liu Y, Liu J, Plante KS, Plante JA, Xie X, Zhang X, Ku Z, An Z, Scharton D, Schindewolf C, Widen SG, Menachery VD, Shi P-Y, Weaver SC. 2022. The N501Y spike substitution enhances SARS-CoV-2 infection and transmission. Nature 602:294–299. doi:10.1038/s41586-021-04245-0 34818667 PMC8900207

[10] Deng X, Garcia-Knight MA, Khalid MM, Servellita V, Wang C, Morris MK, Sotomayor-González A, Glasner DR, Reyes KR, Gliwa AS, et al.. 2021. Transmission, infectivity, and neutralization of a spike L452R SARS-CoV-2 variant. Cell 184:3426–3437. doi:10.1016/j.cell.2021.04.025 33991487 PMC8057738

[11] Greaney AJ, Loes AN, Crawford KHD, Starr TN, Malone KD, Chu HY, Bloom JD. 2021. Comprehensive mapping of mutations in the SARS-CoV-2 receptor-binding domain that affect recognition by polyclonal human plasma antibodies. Cell Host Microbe 29:463–476. doi:10.1016/j.chom.2021.02.003 33592168 PMC7869748

[12] Harvey WT, Carabelli AM, Jackson B, Gupta RK, Thomson EC, Harrison EM, Ludden C, Reeve R, Rambaut A, Peacock SJ, Robertson DL, COVID-19 Genomics UK (COG-UK) Consortium. 2021. SARS-CoV-2 variants, spike mutations and immune escape. Nat Rev Microbiol 19:409–424. doi:10.1038/s41579-021-00573-0 34075212 PMC8167834

[13] WHO. 2022. SARS-CoV-2 variants of concern and variants of interest. Available from: https://www.who.int/en/activities/tracking-SARS-CoV-2-variants

[14] Centers for Disease Control and Prevention. 2022. SARS-CoV-2 variant classifications and definitions. Available from: https://www.cdc.gov/coronavirus/2019-ncov/variants/variant-info.html

[15] Lu L, Mok BW-Y, Chen L, Chan JM-C, Tsang OT-Y, Lam BH-S, Chuang VW-M, Chu AW-H, Chan W-M, Ip JD, Chan BP-C, Zhang R, Yip CC-Y, Cheng VC-C, Chan K-H, Hung IF-N, Yuen K-Y, Chen H, To KK-W. 2021. Neutralization of SARS-CoV-2 Omicron variant by sera from BNT162b2 or CoronaVac vaccine recipients. Clin Infect Dis. doi:10.1101/2021.12.13.21267668 PMC8754807 34915551

[16] Hoffmann M, Krüger N, Schulz S, Cossmann A, Rocha C, Kempf A, Nehlmeier I, Graichen L, Moldenhauer A-S, Winkler MS, Lier M, Dopfer-Jablonka A, Jäck H-M, Behrens GMN, Pöhlmann S. 2022. The Omicron variant is highly resistant against antibody-mediated neutralization: implications for control of the COVID-19 pandemic. Cell 185:447–456. doi:10.1016/j.cell.2021.12.032 35026151 PMC8702401

[17] Domingo E, Holland JJ. 1997. RNA virus mutations and fitness for survival. Annu Rev Microbiol 51:151–178. doi:10.1146/annurev.micro.51.1.151 9343347

[18] Singh D, Yi SV. 2021. On the origin and evolution of SARS-CoV-2. Exp Mol Med 53:537–547. doi:10.1038/s12276-021-00604-z 33864026 PMC8050477

[19] Cyranoski D. 2020. Profile of a killer: the complex biology powering the coronavirus pandemic. Nature 581:22–26. doi:10.1038/d41586-020-01315-7 32367025

[20] Boni MF, Lemey P, Jiang X, Lam TT-Y, Perry BW, Castoe TA, Rambaut A, Robertson DL. 2020. Evolutionary origins of the SARS-CoV-2 sarbecovirus lineage responsible for the COVID-19 pandemic. Nat Microbiol 5:1408–1417. doi:10.1038/s41564-020-0771-4 32724171

[21] Tang X, Wu C, Li X, Song Y, Yao X, Wu X, Duan Y, Zhang H, Wang Y, Qian Z, Cui J, Lu J. 2020. On the origin and continuing evolution of SARS-CoV-2. Natl Sci Rev 7:1012–1023. doi:10.1093/nsr/nwaa036 34676127 PMC7107875

[22] Mascola JR, Graham BS, Fauci AS. 2021. SARS-CoV-2 viral variants—tackling a moving target. JAMA 325:1261–1262. doi:10.1001/jama.2021.2088 33571363

[23] Fiorentini S, Messali S, Zani A, Caccuri F, Giovanetti M, Ciccozzi M, Caruso A. 2021. First detection of SARS-CoV-2 spike protein N501 mutation in Italy in August, 2020. Lancet Infect Dis 21:e147. doi:10.1016/S1473-3099(21)00007-4 33450180 PMC7836831

[24] Zhang W, Davis BD, Chen SS, Sincuir Martinez JM, Plummer JT, Vail E. 2021. Emergence of a novel SARS-CoV-2 variant in Southern California. JAMA 325:1324–1326. doi:10.1001/jama.2021.1612 33571356 PMC7879386

[25] Kandeel M, Mohamed MEM, Abd El-Lateef HM, Venugopala KN, El-Beltagi HS. 2022. Omicron variant genome evolution and phylogenetics. J Med Virol 94:1627–1632. doi:10.1002/jmv.27515 34888894 PMC9015349

[26] Mannar D, Saville JW, Zhu X, Srivastava SS, Berezuk AM, Tuttle KS, Marquez AC, Sekirov I, Subramaniam S. 2022. SARS-CoV-2 Omicron variant: antibody evasion and cryo-EM structure of spike protein–ACE2 complex. Science 375:760–764. doi:10.1126/science.abn7760 35050643 PMC9799367

[27] Araf Y, Akter F, Tang Y-D, Fatemi R, Parvez MSA, Zheng C, Hossain MG. 2022. Omicron variant of SARS-CoV-2: genomics, transmissibility, and responses to current COVID-19 vaccines. J Med Virol 94:1825–1832. doi:10.1002/jmv.27588 35023191 PMC9015557

[28] Shrestha LB, Foster C, Rawlinson W, Tedla N, Bull RA. 2022. Evolution of the SARS-CoV-2 Omicron variants BA.1 to BA.5: implications for immune escape and transmission. Rev Med Virol 32:e2381. doi:10.1002/rmv.2381 35856385 PMC9349777

[29] Yaniv K, Ozer E, Shagan M, Paitan Y, Granek R, Kushmaro A. 2022. Managing an evolving pandemic: cryptic circulation of the delta variant during the Omicron rise. Sci Total Environ 836:155599. doi:10.1016/j.scitotenv.2022.155599 35504376 PMC9055682

[30] Wang L, Cheng G. 2022. Sequence analysis of the emerging SARS-CoV-2 variant Omicron in South Africa. J Med Virol 94:1728–1733. doi:10.1002/jmv.27516 34897752

[31] Kupferschmidt K. 2021. Where did ‘weird’ Omicron come from? Science 374:1179. doi:10.1126/science.acx9738 34855502

[32] Karim F, Moosa M, Gosnell B, Cele S, Giandhari J, Pillay S, Tegally H, Wilkinson E, San J, Msomi N, Mlisana K, Khan K, Bernstein M, Manickchund N, Singh L, Ramphal U, Hanekom W, Lessells R, Sigal A, de Oliveira T, COMMIT-KZN Team. 2021. Persistent SARS-CoV-2 infection and intra-host evolution in association with advanced HIV infection. Infect Dis. doi:10.1101/2021.06.03.21258228

[33] Wei C, Shan K-J, Wang W, Zhang S, Huan Q, Qian W. 2021. Evidence for a mouse origin of the SARS-CoV-2 Omicron variant. J Genet Genomics 48:1111–1121. doi:10.1016/j.jgg.2021.12.003 34954396 PMC8702434

[34] Xu Y, Wu C, Cao X, Gu C, Liu H, Jiang M, Wang X, Yuan Q, Wu K, Liu J, Wang D, He X, Wang X, Deng S-J, Xu HE, Yin W. 2022. Structural and biochemical mechanism for increased infectivity and immune evasion of Omicron BA.2 variant compared to BA.1 and their possible mouse origins. Cell Res 32:609–620. doi:10.1038/s41422-022-00672-4 35641567 PMC9152305

[35] Han P, Li L, Liu S, Wang Q, Zhang D, Xu Z, Han P, Li X, Peng Q, Su C, Huang B, Li D, Zhang R, Tian M, Fu L, Gao Y, Zhao X, Liu K, Qi J, Gao GF, Wang P. 2022. Receptor binding and complex structures of human ACE2 to spike RBD from Omicron and delta SARS-CoV-2. Cell 185:630–640. doi:10.1016/j.cell.2022.01.001 35093192 PMC8733278

[36] Xia B, Wang Y, Pan X, Cheng X, Ji H, Zuo X, Jiang H, Li J, Gao Z. 2022. Why is the SARS-CoV-2 Omicron variant milder? Innovation (Camb) 3:100251. doi:10.1016/j.xinn.2022.100251 35497020 PMC9040510

[37] Peacock TP, Goldhill DH, Zhou J, Baillon L, Frise R, Swann OC, Kugathasan R, Penn R, Brown JC, Sanchez-David RY, Braga L, Williamson MK, Hassard JA, Staller E, Hanley B, Osborn M, Giacca M, Davidson AD, Matthews DA, Barclay WS. 2021. The furin cleavage site in the SARS-CoV-2 spike protein is required for transmission in ferrets. Nat Microbiol 6:899–909. doi:10.1038/s41564-021-00908-w 33907312 PMC7619196

[38] Ambati BK, Varshney A, Lundstrom K, Palú G, Uhal BD, Uversky VN, Brufsky AM. 2022. MSH3 homology and potential recombination link to SARS-CoV-2 furin cleavage site. Front Virol 2. doi:10.3389/fviro.2022.834808 PMC11340822 39176223

[39] Hossain MG, Tang YD, Akter S, Zheng C. 2022. Roles of the polybasic furin cleavage site of spike protein in SARS-CoV-2 replication, pathogenesis, and host immune responses and vaccination. J Med Virol 94:1815–1820. doi:10.1002/jmv.27539 34936124

[40] Levin D, Raab N, Pinto Y, Rothschild D, Zanir G, Godneva A, Mellul N, Futorian D, Gal D, Leviatan S, Zeevi D, Bachelet I, Segal E. 2021. Diversity and functional landscapes in the microbiota of animals in the wild. Science 372:eabb5352. doi:10.1126/science.abb5352 33766942

[41] Lloyd-Price J, Abu-Ali G, Huttenhower C. 2016. The healthy human microbiome. Genome Med 8:51. doi:10.1186/s13073-016-0307-y 27122046 PMC4848870

[42] Yi X, Gao J, Wang Z. 2022. The human lung microbiome—a hidden link between microbes and human health and diseases. iMeta 1. doi:10.1002/imt2.33 PMC10989958 38868714

[43] Milani C, Duranti S, Bottacini F, Casey E, Turroni F, Mahony J, Belzer C, Delgado Palacio S, Arboleya Montes S, Mancabelli L, Lugli GA, Rodriguez JM, Bode L, de Vos W, Gueimonde M, Margolles A, van Sinderen D, Ventura M. 2017. The first microbial colonizers of the human gut: composition, activities, and health implications of the infant gut microbiota. Microbiol Mol Biol Rev 81:e00036-17. doi:10.1128/MMBR.00036-17 PMC5706746 29118049

[44] Yatsunenko T, Rey FE, Manary MJ, Trehan I, Dominguez-Bello MG, Contreras M, Magris M, Hidalgo G, Baldassano RN, Anokhin AP, Heath AC, Warner B, Reeder J, Kuczynski J, Caporaso JG, Lozupone CA, Lauber C, Clemente JC, Knights D, Knight R, Gordon JI. 2012. Human gut microbiome viewed across age and geography. Nature 486:222–227. doi:10.1038/nature11053 22699611 PMC3376388

[45] Suzuki TA, Ley RE. 2020. The role of the microbiota in human genetic adaptation. Science 370. doi:10.1126/science.aaz6827 33273073

[46] Man WH, de Steenhuijsen Piters WAA, Bogaert D. 2017. The microbiota of the respiratory tract: gatekeeper to respiratory health. Nat Rev Microbiol 15:259–270. doi:10.1038/nrmicro.2017.14 28316330 PMC7097736

[47] Patel KP, Patel PA, Vunnam RR, Hewlett AT, Jain R, Jing R, Vunnam SR. 2020. Gastrointestinal, hepatobiliary, and pancreatic manifestations of COVID-19. J Clin Virol 128:104386. doi:10.1016/j.jcv.2020.104386 32388469 PMC7189193

[48] Mao R, Qiu Y, He J-S, Tan J-Y, Li X-H, Liang J, Shen J, Zhu L-R, Chen Y, Iacucci M, Ng SC, Ghosh S, Chen M-H. 2020. Manifestations and prognosis of gastrointestinal and liver involvement in patients with COVID-19: a systematic review and meta-analysis. Lancet Gastroenterol Hepatol 5:667–678. doi:10.1016/S2468-1253(20)30126-6 32405603 PMC7217643

[49] Zuo T, Zhang F, Lui GCY, Yeoh YK, Li AYL, Zhan H, Wan Y, Chung ACK, Cheung CP, Chen N, Lai CKC, Chen Z, Tso EYK, Fung KSC, Chan V, Ling L, Joynt G, Hui DSC, Chan FKL, Chan PKS, Ng SC. 2020. Alterations in gut microbiota of patients with COVID-19 during time of hospitalization. Gastroenterology 159:944–955. doi:10.1053/j.gastro.2020.05.048 32442562 PMC7237927

[50] Villapol S. 2020. Gastrointestinal symptoms associated with COVID-19: impact on the gut microbiome. Transl Res 226:57–69. doi:10.1016/j.trsl.2020.08.004 32827705 PMC7438210

[51] Liang W, Feng Z, Rao S, Xue X, Lin Z, Zhang Q, Qi W. 2020. Diarrhoea may be underestimated: a missing link in 2019 novel coronavirus. Gut 69:1141–1143. doi:10.1136/gutjnl-2020-320832 32102928

[52] Yeoh YK, Zuo T, Lui GC-Y, Zhang F, Liu Q, Li AY, Chung AC, Cheung CP, Tso EY, Fung KS, Chan V, Ling L, Joynt G, Hui DS-C, Chow KM, Ng SSS, Li TC-M, Ng RW, Yip TC, Wong GL-H, Chan FK, Wong CK, Chan PK, Ng SC. 2021. Gut microbiota composition reflects disease severity and dysfunctional immune responses in patients with COVID-19. Gut 70:698–706. doi:10.1136/gutjnl-2020-323020 33431578 PMC7804842

[53] Liu Q, Mak JWY, Su Q, Yeoh YK, Lui GC-Y, Ng SSS, Zhang F, Li AYL, Lu W, Hui DS-C, Chan PK, Chan FKL, Ng SC. 2022. Gut microbiota dynamics in a prospective cohort of patients with post-acute COVID-19 syndrome. Gut 71:544–552. doi:10.1136/gutjnl-2021-325989 35082169

[54] Cao J, Wang C, Zhang Y, Lei G, Xu K, Zhao N, Lu J, Meng F, Yu L, Yan J, Bai C, Zhang S, Zhang N, Gong Y, Bi Y, Shi Y, Chen Z, Dai L, Wang J, Yang P. 2021. Integrated gut virome and bacteriome dynamics in COVID-19 patients. Gut Microbes 13:1–21. doi:10.1080/19490976.2021.1887722 PMC7946006 33678150

[55] Donati Zeppa S, Agostini D, Piccoli G, Stocchi V, Sestili P. 2020. Gut microbiota status in COVID-19: an unrecognized player? Front Cell Infect Microbiol 10. doi:10.3389/fcimb.2020.576551 PMC7725702 33324572

[56] Chen Y, Gu S, Chen Y, Lu H, Shi D, Guo J, Wu W-R, Yang Y, Li Y, Xu K-J, Ding C, Luo R, Huang C, Yu L, Xu M, Yi P, Liu J, Tao J-J, Zhang H, Lv L, Wang B, Sheng J, Li L. 2022. Six-month follow-up of gut microbiota richness in patients with COVID-19. Gut 71:222–225. doi:10.1136/gutjnl-2021-324090 33833065 PMC8666823

[57] Duffy S, Shackelton LA, Holmes EC. 2008. Rates of evolutionary change in viruses: patterns and determinants. Nat Rev Genet 9:267–276. doi:10.1038/nrg2323 18319742

[58] Koyama T, Platt D, Parida L. 2020. Variant analysis of SARS-CoV-2 genomes. Bull World Health Organ 98:495–504. doi:10.2471/BLT.20.253591 32742035 PMC7375210

[59] Wang D, Jiang A, Feng J, Li G, Guo D, Sajid M, Wu K, Zhang Q, Ponty Y, Will S, Liu F, Yu X, Li S, Liu Q, Yang X-L, Guo M, Li X, Chen M, Shi Z-L, Lan K, Chen Y, Zhou Y. 2021. The SARS-CoV-2 subgenome landscape and its novel regulatory features. Mol Cell 81:2135–2147. doi:10.1016/j.molcel.2021.02.036 33713597 PMC7927579

[60] Negroni M, Buc H. 2001. Retroviral recombination: what drives the switch? Nat Rev Mol Cell Biol 2:151–155. doi:10.1038/35052098 11252957

[61] Sola I, Almazán F, Zúñiga S, Enjuanes L. 2015. Continuous and discontinuous RNA synthesis in coronaviruses. Annu Rev Virol 2:265–288. doi:10.1146/annurev-virology-100114-055218 26958916 PMC6025776

[62] Chrisman BS, Paskov K, Stockham N, Tabatabaei K, Jung J-Y, Washington P, Varma M, Sun MW, Maleki S, Wall DP. 2021. Indels in SARS-CoV-2 occur at template-switching hotspots. BioData Min 14:20. doi:10.1186/s13040-021-00251-0 33743803 PMC7980745

[63] Sztuba-Solińska J, Urbanowicz A, Figlerowicz M, Bujarski JJ. 2011. RNA-RNA recombination in plant virus replication and evolution. Annu Rev Phytopathol 49:415–443. doi:10.1146/annurev-phyto-072910-095351 21529157

[64] Negroni M, Buc H. 2001. Mechanisms of retroviral recombination. Annu Rev Genet 35:275–302. doi:10.1146/annurev.genet.35.102401.090551 11700285

[65] Chen X, Liao B, Cheng L, Peng X, Xu X, Li Y, Hu T, Li J, Zhou X, Ren B. 2020. The microbial coinfection in COVID-19. Appl Microbiol Biotechnol 104:7777–7785. doi:10.1007/s00253-020-10814-6 32780290 PMC7417782

[66] Rawson TM, Moore LSP, Zhu N, Ranganathan N, Skolimowska K, Gilchrist M, Satta G, Cooke G, Holmes A. 2020. Bacterial and fungal coinfection in individuals with coronavirus: a rapid review to support COVID-19 antimicrobial prescribing. Clin Infect Dis 71:2459–2468. doi:10.1093/cid/ciaa530 32358954 PMC7197596

[67] Bengoechea JA, Bamford CG. 2020. SARS-CoV-2, bacterial co-infections, and AMR: the deadly trio in COVID-19? EMBO Mol Med 12:e12560. doi:10.15252/emmm.202012560 32453917 PMC7283846

[68] Wendisch D, Dietrich O, Mari T, von Stillfried S, Ibarra IL, Mittermaier M, Mache C, Chua RL, Knoll R, Timm S, et al.. 2021. SARS-CoV-2 infection triggers profibrotic macrophage responses and lung fibrosis. Cell 184:6243–6261. doi:10.1016/j.cell.2021.11.033 34914922 PMC8626230

[69] Mirzaei R, Goodarzi P, Asadi M, Soltani A, Aljanabi HAA, Jeda AS, Dashtbin S, Jalalifar S, Mohammadzadeh R, Teimoori A, Tari K, Salari M, Ghiasvand S, Kazemi S, Yousefimashouf R, Keyvani H, Karampoor S. 2020. Bacterial co-infections with SARS-CoV-2. IUBMB Life 72:2097–2111. doi:10.1002/iub.2356 32770825 PMC7436231

[70] Farrell JM, Zhao CY, Tarquinio KM, Brown SP. 2021. Causes and consequences of COVID-19-associated bacterial infections. Front Microbiol 12:682571. doi:10.3389/fmicb.2021.682571 34354682 PMC8329088

[71] Moore MD, Jaykus LA. 2018. Virus-bacteria interactions: implications and potential for the applied and agricultural sciences. Viruses 10:61. doi:10.3390/v10020061 29393885 PMC5850368

[72] Neurath MF, Überla K, Ng SC. 2021. Gut as viral reservoir: lessons from gut viromes, HIV and COVID-19. Gut 70:1605–1608. doi:10.1136/gutjnl-2021-324622 33903146

[73] Lamers MM, Beumer J, van der Vaart J, Knoops K, Puschhof J, Breugem TI, Ravelli RBG, Paul van Schayck J, Mykytyn AZ, Duimel HQ, van Donselaar E, Riesebosch S, Kuijpers HJH, Schipper D, van de Wetering WJ, de Graaf M, Koopmans M, Cuppen E, Peters PJ, Haagmans BL, Clevers H. 2020. SARS-CoV-2 productively infects human gut enterocytes. Science 369:50–54. doi:10.1126/science.abc1669 32358202 PMC7199907

[74] Samson JE, Magadán AH, Sabri M, Moineau S. 2013. Revenge of the phages: defeating bacterial defences. Nat Rev Microbiol 11:675–687. doi:10.1038/nrmicro3096 23979432

[75] Erickson AK, Jesudhasan PR, Mayer MJ, Narbad A, Winter SE, Pfeiffer JK. 2018. Bacteria facilitate enteric virus co-infection of mammalian cells and promote genetic recombination. Cell Host Microbe 23:77–88. doi:10.1016/j.chom.2017.11.007 29290575 PMC5764776

[76] DeLong JP, Van Etten JL, Al-Ameeli Z, Agarkova IV, Dunigan DD. 2023. The consumption of viruses returns energy to food chains. Proc Natl Acad Sci USA 120:e2215000120. doi:10.1073/pnas.2215000120 36574690 PMC9910503

[77] Simon-Loriere E, Holmes EC. 2011. Why do RNA viruses recombine? Nat Rev Microbiol 9:617–626. doi:10.1038/nrmicro2614 21725337 PMC3324781

[78] Sanjuán R, Domingo-Calap P. 2016. Mechanisms of viral mutation. Cell Mol Life Sci 73:4433–4448. doi:10.1007/s00018-016-2299-6 27392606 PMC5075021

[79] Chen R, Holmes EC. 2006. Avian influenza virus exhibits rapid evolutionary dynamics. Mol Biol Evol 23:2336–2341. doi:10.1093/molbev/msl102 16945980

[80] Carvalho VL, Long MT. 2021. Insect-specific viruses: an overview and their relationship to arboviruses of concern to humans and animals. Virology 557:34–43. doi:10.1016/j.virol.2021.01.007 33631523

[81] Gao F, Bailes E, Robertson DL, Chen Y, Rodenburg CM, Michael SF, Cummins LB, Arthur LO, Peeters M, Shaw GM, Sharp PM, Hahn BH. 1999. Origin of HIV-1 in the chimpanzee pan troglodytes troglodytes. Nature 397:436–441. doi:10.1038/17130 9989410

[82] Prasad A, Sharma N, Hari-Gowthem G, Muthamilarasan M, Prasad M. 2020. Tomato yellow leaf curl virus: impact, challenges, and management. Trends Plant Sci 25:897–911. doi:10.1016/j.tplants.2020.03.015 32371058

[83] Jackson B, Boni MF, Bull MJ, Colleran A, Colquhoun RM, Darby AC, Haldenby S, Hill V, Lucaci A, McCrone JT, Nicholls SM, O’Toole Á, Pacchiarini N, Poplawski R, Scher E, Todd F, Webster HJ, Whitehead M, Wierzbicki C, COVID-19 Genomics UK (COG-UK) Consortium, Loman NJ, Connor TR, Robertson DL, Pybus OG, Rambaut A. 2021. Generation and transmission of interlineage recombinants in the SARS-CoV-2 pandemic. Cell 184:5179–5188. doi:10.1016/j.cell.2021.08.014 34499854 PMC8367733

[84] Dolan PT, Whitfield ZJ, Andino R. 2018. Mechanisms and concepts in RNA virus population dynamics and evolution. Annu Rev Virol 5:69–92. doi:10.1146/annurev-virology-101416-041718 30048219 PMC13283306

[85] Cao Y, Jian F, Wang J, Yu Y, Song W, Yisimayi A, Wang J, An R, Chen X, Zhang N, et al.. 2023. Imprinted SARS-CoV-2 humoral immunity induces convergent Omicron RBD evolution. Nature 614:521–529. doi:10.1038/s41586-022-05644-7 36535326 PMC9931576

[86] Zhang F, Lau RI, Liu Q, Su Q, Chan FKL, Ng SC. 2023. Gut microbiota in COVID-19: key microbial changes, potential mechanisms and clinical applications. Nat Rev Gastroenterol Hepatol 20:323–337. doi:10.1038/s41575-022-00698-4 36271144 PMC9589856

[87] Tsuchiya H. 2023. The oral cavity potentially serving as a reservoir for SARS-CoV-2 but not necessarily facilitating the spread of COVID-19 in dental practice. Eur J Dent 17:310–318. doi:10.1055/s-0042-1757909 36539210 PMC10329531

[88] Gang J, Wang H, Xue X, Zhang S. 2022. Microbiota and COVID-19: long-term and complex influencing factors. Front Microbiol 13:963488. doi:10.3389/fmicb.2022.963488 36033885 PMC9417543

[89] De R, Dutta S. 2022. Role of the microbiome in the pathogenesis of COVID-19. Front Cell Infect Microbiol 12:736397. doi:10.3389/fcimb.2022.736397 35433495 PMC9009446

[90] Dragelj J, Mroginski MA, Ebrahimi KH. 2021. Hidden in plain sight: natural products of commensal microbiota as an environmental selection pressure for the rise of new variants of SARS-CoV-2. Chembiochem 22:2946–2950. doi:10.1002/cbic.202100346 34265150 PMC8427076

