# Supplementary material for: The human microbiota is a beneficial reservoir for SARS-CoV-2 mutations
Source: mBio. 2024 Mar 26;15(5):e03187-23. doi: 10.1128/mbio.03187-23 (PMC11237538; doi:10.1128/mbio.03187-23)
Supplement: Supplemental material — Fig. S1-S6 and captions to supplemental tables. [file mbio.03187-23-s0007.docx]

**Supplemental Figures and Tables：**

**Table S1. Frequency of all mutations in 5 VOC variants.** All mutations have frequencies above 90%, which were referred to CNCB-NGDC RCoV19, NCBI Coronavirus Resource, and EBI coronavirus database.

**Table S2. The HFs sequences of the spike, envelop, and membrane proteins. 7**aa HFs were composed of mutation and its adjacent 6 amino acids (aa).

**Table S3-1. Original data for HFs on spike protein.**

**Table S3-2. Original data for HFs on envelope protein.**

**Table S3-3. Original data for HFs on membrane protein.**

**Table S4. Original data for 9aa HFs on spike, envelope, and membrane proteins.**

**Table S5.** **Original data for HFs on Omicron** **subvariants, including BA.5.2, BF.7, BQ.1.1 and XBB.**

**Table S6. The primer list for RdRp extension assays.**


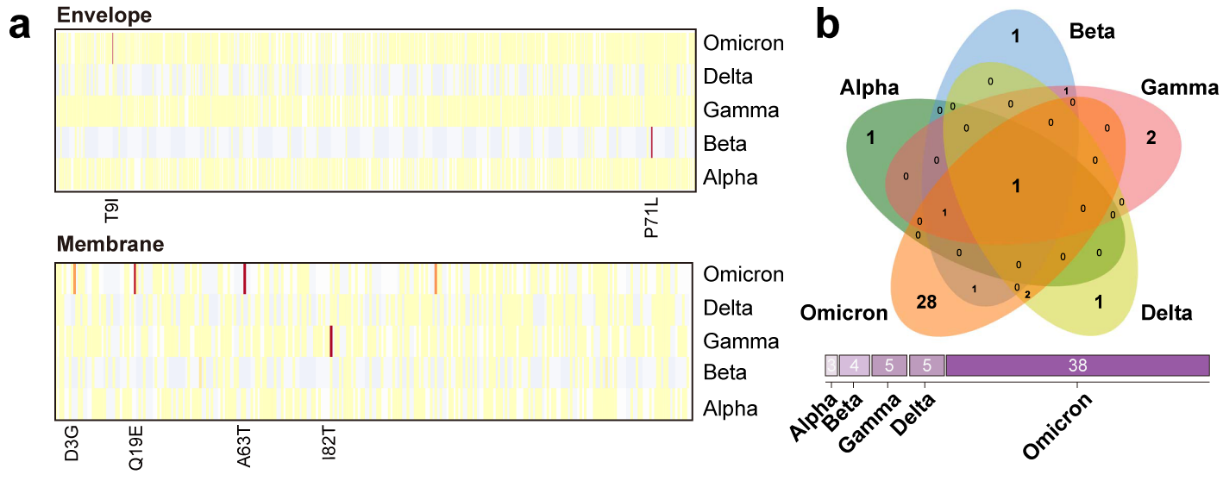


**Fig.S1 Beneficial mutaions of VOCs.**

**a,** The mutation probability of envelope and membrane protein mutations in all VOCs. **b,** Comparison of highly frequent mutations in different VOCs.

**
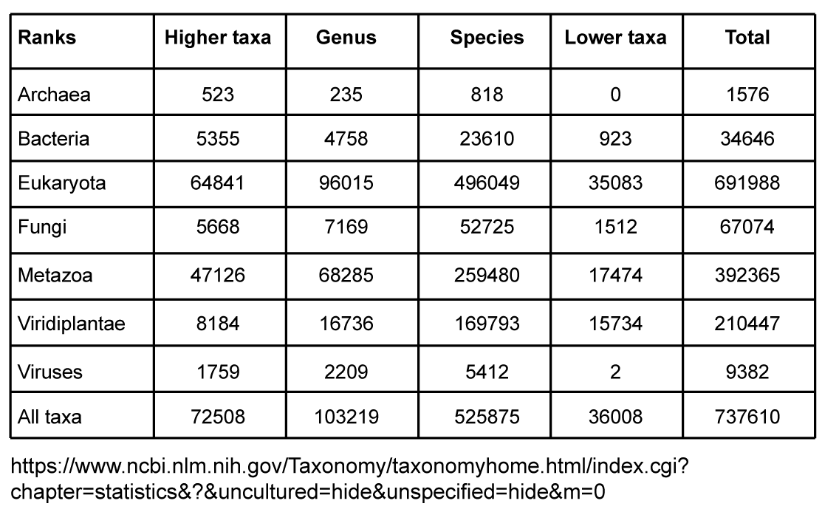
**

**Fig. S2. Species taxonomy in NCBI database.** The database currently represents about 10% of described species of life on the planet (October 2022).

**
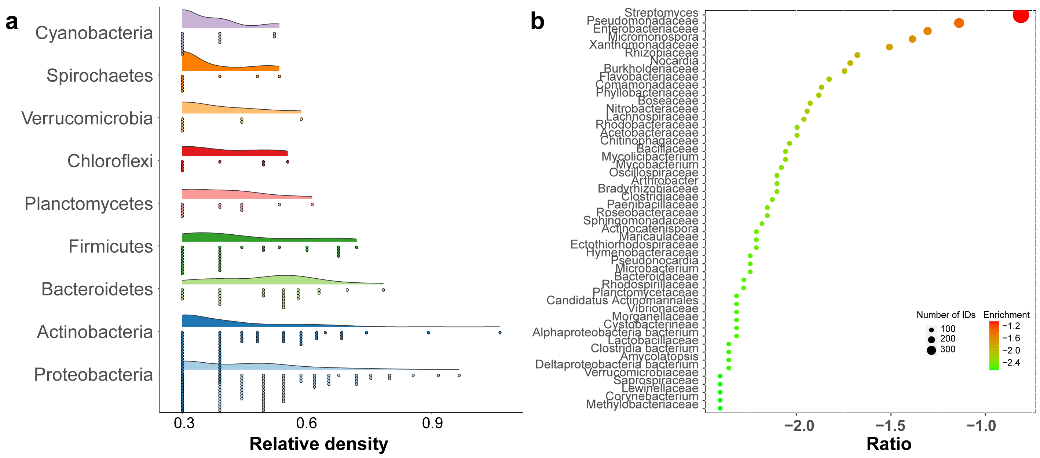
**

**Fig. S3. The HFs composition analysis of obtained bacteria at family and Genus levels.**

**a,** Composition of top bacteria phylum at the family level. The raindrops represent the family types of each phylum. The violin plot represents the relative density of different families contained in different phyla in the total number and the individual data points represent the number of genera contained in each family. **b,** Enrichment analysis of the obtained bacteria at the family level. Color-coded with log10 transformed of family percentage.


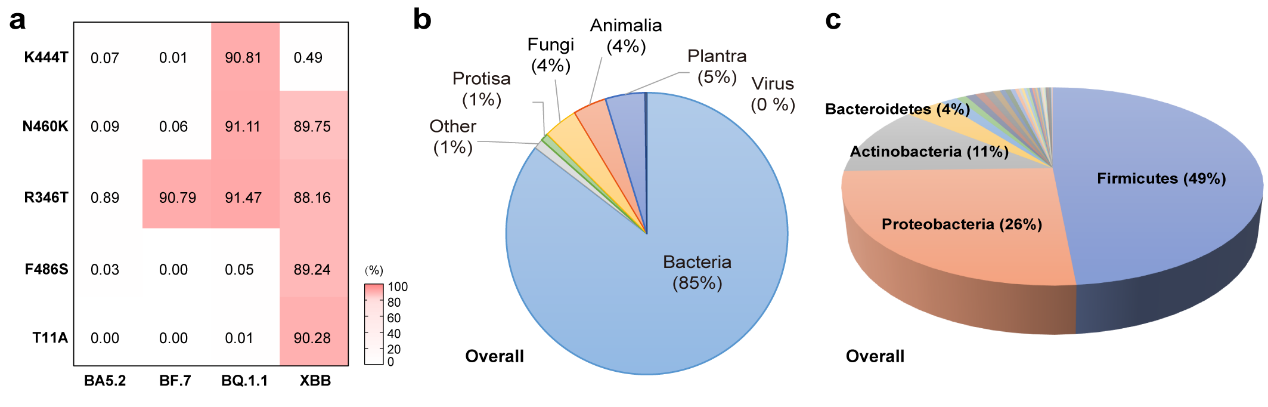


**Fig. S4.** **Homologous fragments (HFs) alignment of new mutation fragments on the Omicron subvariants.**

**a,** Heatmap of 5 beneficial mutations frequencies in Omicron subvariants. **b,** Pie chart representations of HFs Kingdoms from overall (bigger one), and four mutations on spike (S), one mutation on envelope (E) (smaller five). The species proportions in HFs and NCBI databases. **c,** Pie chart representations of the obtained bacteria, all items at the phylum level.

**
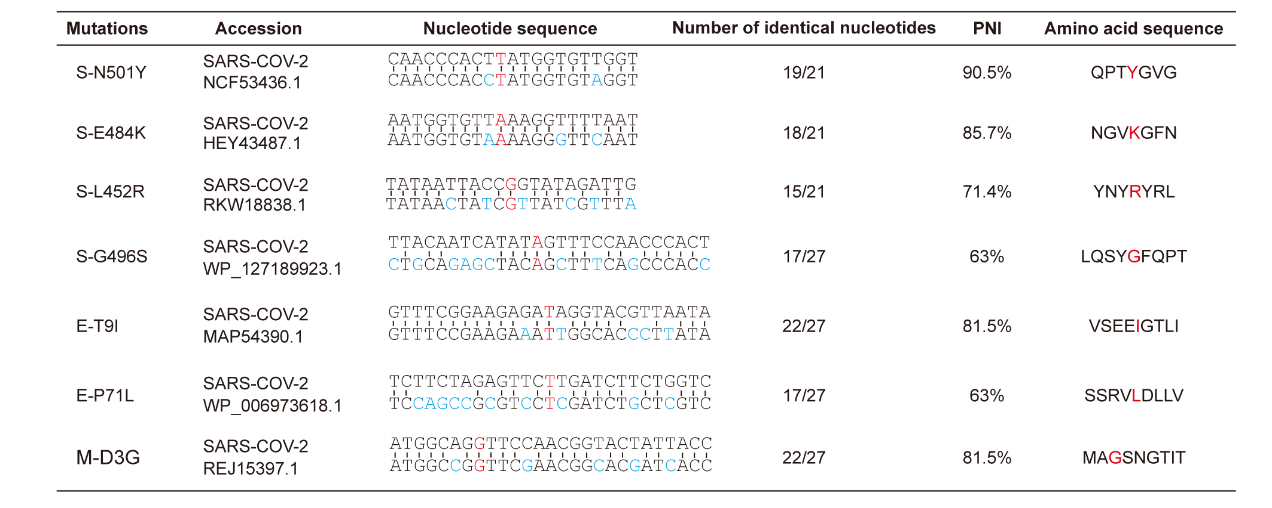
**

**Fig. S5. The alignment of nucleotide sequences of bacterial mRNAs that encode the same amino acid sequences with viral RNAs**, including the mutations from S protein (N501Y, E484K, L452R, and G496S), E protein (T9I, P71L) and M protein (D3G). Red represents a mutated base or amino acid, and blue represents inconsistent bases**.** PNI is the percent nucleotide identity**.**

**
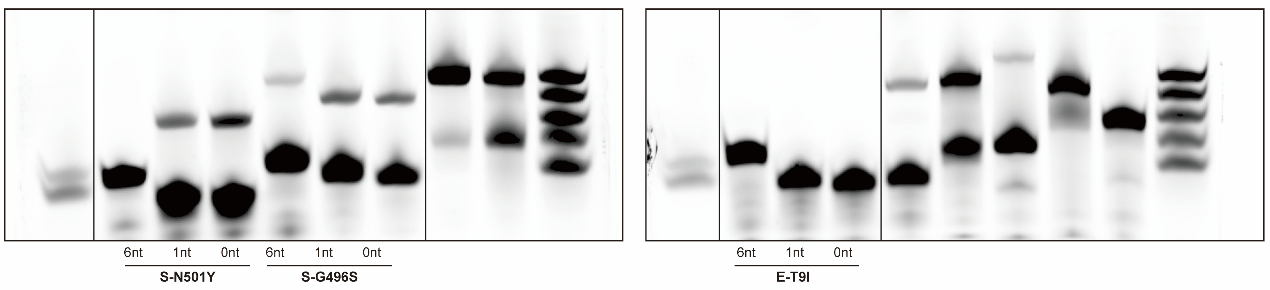
**

**Fig. S6. SARS-CoV-2 RdRp introduces bacterial mRNA nucleotide mutations into viral RNAs through homologous recombination.**

The original gel data for Figure 4. It is the mismatch extension experiments in vitro, N501Y, G496S (Left), and T9I mutations (Right) for RdRp extension.
